# Tracking plant preference for higher‐quality mycorrhizal symbionts under varying CO_2_ conditions over multiple generations

**DOI:** 10.1002/ece3.3635

**Published:** 2017-11-23

**Authors:** Gijsbert D. A. Werner, Yeling Zhou, Corné M. J. Pieterse, E. Toby Kiers

**Affiliations:** ^1^ Department of Ecological Science Vrije Universiteit Amsterdam Amsterdam The Netherlands; ^2^ Department of Zoology University of Oxford Oxford UK; ^3^ Plant‐Microbe Interactions Department of Biology Utrecht University Utrecht The Netherlands

**Keywords:** context‐dependence, global change, mutualism, rewards, sanction

## Abstract

The symbiosis between plants and root‐colonizing arbuscular mycorrhizal (AM) fungi is one of the most ecologically important examples of interspecific cooperation in the world. AM fungi provide benefits to plants; in return plants allocate carbon resources to fungi, preferentially allocating more resources to higher‐quality fungi. However, preferential allocations from plants to symbionts may vary with environmental context, particularly when resource availability affects the relative value of symbiotic services. We ask how differences in atmospheric CO_2_‐levels influence root colonization dynamics between AMF species that differ in their quality as symbiotic partners. We find that with increasing CO_2_‐conditions and over multiple plant generations, the more beneficial fungal species is able to achieve a relatively higher abundance. This suggests that increasing atmospheric carbon supply enables plants to more effectively allocate carbon to higher‐quality mutualists, and over time helps reduce lower‐quality AM abundance. Our results illustrate how environmental context may affect the extent to which organisms structure interactions with their mutualistic partners and have potential implications for mutualism stability and persistence under global change.

## 1 INTRODUCTION

Organisms across the tree of life rely on symbiotic associations with other organisms to obtain resources and services that would otherwise be inaccessible or costly to acquire (Douglas, 2010; Leigh, 2010). Yet, the outcome of symbiotic partnerships can be highly context‐dependent, varying from strongly beneficial to both partners (mutualism) to a net fitness cost for one of the partners (parasitism) (Bronstein, 1994; Chamberlain, Bronstein, & Rudgers, 2014; Hoeksema et al., 2010). A key factor driving such context‐dependence is variation in the environmental availability of the symbiotically provided resources (Bever, 2015; Konvalinková & Jansa, 2016; de Mazancourt & Schwartz, 2010; Shantz & Burkepile, 2014; Weese et al., 2015). For instance, if a legume grows in a high‐nitrogen habitat, it can be cheaper to acquire nitrogen from the soil directly, than to invest carbon in nitrogen‐fixing rhizobial symbionts (Heath & Tiffin, 2007; Lau et al., 2012). Context can also affect symbiotic outcomes through variation in partner quality (Denison & Kiers, 2011; Ness, Morris, & Bronstein, 2006). Partners can vary in the benefits they provide, and in some cases low‐quality partners can even have negative effects on host growth (Ghoul, Griffin, & West, 2014; Hart et al., 2012; Sachs et al., 2010). In order to limit the impact of such low‐quality partners, many organisms have evolved mechanisms, including various forms of partner choice, rewards or sanctions that lead to preferential associations with higher‐quality partners (Chomicki et al., 2016; Gubry‐Rangin, Garcia, & Bena, 2010; Jander & Herre, 2016; Kaltenpoth et al., 2014; Wang, Dunn, & Sun, 2014). However, we know little about how these mechanisms are influenced by environmental context. Can a plant host's ability to favor higher‐quality partners or to discriminate against low‐quality partners be impacted by changes in the environment?

The symbiosis between plants and arbuscular mycorrhizal (AM) fungi is emerging as an important system to study how context influences symbiotic partnerships (Chandrasekaran et al., 2014; Hoeksema et al., 2010; Johnson, 2010; Neuhauser & Fargione, 2004; Ossler, Zielinski, & Heath, 2015). Plants invest in their AM fungal partners by providing them with carbon, while the fungi can benefit their hosts by providing soil minerals, primarily phosphorus (Parniske, 2008). There is accumulating evidence that both host plants and fungi can detect differences in partner contributions, and preferentially allocate carbon or soil minerals to those providing higher benefits (Bever et al., 2009; Fellbaum et al., 2012, 2014; Hammer et al., 2011; Kiers et al., 2011). AM fungi are characterized by their localized intracellular structures (arbuscules) where nutrient exchange takes place. This potentially allows for preferential allocation to more beneficial AM fungi by facilitating directed transfer of carbon to specific fungi, and by enabling plant control over arbuscule life span through cell‐specific nutrient supply (Gutjahr & Parniske, 2013; Kiers et al., 2016; Luginbuehl & Oldroyd, 2017). Over evolutionary time, such reciprocal rewarding mechanisms might lead to a coevolutionary process where both partners maintain investment in the other partner (Bever, 2015; Kiers et al., 2011), as is generally observed in plant AM fungal interactions (Chaudhary et al., 2016; Hoeksema et al., 2010). While these preferential allocation mechanisms are thought to help reduce conflict and stabilize mutualistic relationships (Argüello et al., 2016; Bever, 2015; Kiers et al., 2011, 2016), the role of context in allocating benefits to interacting partners is not well understood.

Theory predicts that individuals should be able to plastically respond to variable conditions, particularly those that affect the relative value of the exchanged resource, allowing them to maximize symbiotic benefit, or similarly, to reduce cost of parasitism (Bever, 2015; Cowden & Peterson, 2009; Ji et al., 2013; Kummel & Salant, 2006; Wyatt et al., 2014). For example, experimental work has revealed that plant preferential allocations decline with increasing soil phosphorus and shading (Ji & Bever, 2016; Zheng et al., 2015), but the impact of other key factors, such as atmospheric CO_2_‐concentrations, has yet to be tested. There are three potential effects of increasing CO_2_‐levels on plant preferential selection of AM fungal symbionts: (1) Its efficiency and strength could be increased, resulting in higher abundance of higher‐quality AM fungi (Bever, 2015; Johnson et al., 2013; Wyatt et al., 2014), (2) it could become less important for host plants to stringently allocate photosynthates, as availability of carbon increases with increased CO_2_‐levels, resulting in relatively higher abundance of low‐quality AM fungi (Golubski & Klausmeier, 2010; Kiers & van der Heijden, 2006), or (3) CO_2_‐levels could have no effect on plant relative allocations, for instance because plant allocations respond primarily to fungal identity and not quality.

We wanted to test if CO_2‐_levels mediate changes in host carbon allocations to fungal partners that vary in the benefit they provide to their host plants. CO_2_‐concentration is one of the primary drivers of carbon availability. Over the last centuries, CO_2_‐levels increased from an estimated 278 ppm in the 18^th^ century to 406 ppm in March 2017 (Hartmann et al., 2013; National Oceanic and Atmospheric Administration, US Department of Commerce, url: https://climate.nasa.gov/vital-signs/carbon-dioxide/). Such an increase has been credited with reducing plant carbon limitation (Ainsworth & Long, 2005; Reich et al., 2006) and potentially increasing total AM root colonization and modifying fungal competition dynamics (Alberton, Kuyper, & Gorissen, 2005; Drigo et al., 2010, 2013; Fortuna et al., 2012; Treseder, 2004). In contrast, over longer time scales, CO_2_‐levels were considerably lower than ambient, reaching as low as 180 ppm in the late Pleistocene (~17.5 Ka ago) (Temme et al., 2013). These glacial atmospheric CO_2_‐levels are thought to have increased plant carbon limitation and reduced the benefit of interaction with AM fungi (Becklin, Mullinix, & Ward, 2016; Field et al., 2012). As obligate biotrophs, AM fungi can only obtain carbon from host plants (Parniske, 2008). This means they have access to additional (or reduced) carbon, only through plant allocations, and not directly.

Our aim was to determine if shifts in CO_2_‐level affect the extent to which plants favor higher‐quality AM fungal partners, and how this affects the spread of low‐quality partners across multiple host‐plant generations. To address these questions, we ran a multigenerational study to understand the effects of depressed and elevated (relative to the present ambient) CO_2_‐levels on the success of two closely related AM species that vary in the benefits they provide (Kiers et al., 2011). While previous work has shown broad‐pattern shifts in AM fungal communities under varying CO_2_‐levels (e.g., from *Acaulosporaceae* and *Gigasporaceae* to *Glomeraceae* (Klironomos et al., 2005; Drigo et al., 2010; Cotton et al., 2015), these studies did not test how environmental change‐mediated multigenerational shifts across specific AM species that differ in terms of their quality as symbiotic partners but are otherwise closely related. Here, we tested the three potential scenarios by growing host plants in depressed (~160 ppm), ambient (~490 ppm), or elevated (~750 ppm) atmospheric CO_2_‐levels, and analyzing the effect on host growth and the relative abundance of two competing AM species (both *Glomeraceae,* but differing in the quality of benefits they provide to their host plants) over multiple plant generations. We performed a multigenerational experiment, because CO_2_ effects could be weak and a potential impact on the relative success of a higher‐quality AM fungus might take time to become detectable (Klironomos et al., 2005; Wyatt et al., 2014).

## 2 MATERIALS AND METHODS

### 2.1 Experimental design

We inoculated *Medicago truncatula* Gaertn. (courtesy of Prof. B. Hause, Leibniz Institute of Plant Biochemistry, Halle, Germany) seedlings with one of four mycorrhizal treatments (1) a monoculture of *Glomus aggregatum*, (2) a monoculture of *Rhizophagus irregularis*, (formerly known as *Glomus intraradices* [Krüger et al., 2012]), (3) 1:1 mixture of both species, or (4) without AMF (negative control). Previous research had shown that *R. irregularis* is a higher‐quality symbiont that is more beneficial to host plants, while *G. aggregatum* employs a less cooperative hoarding strategy (Knegt et al., 2016), which stores substantially more of its phosphorus in a poly‐P form inaccessible to plants and results in depressed growth of the host plant (Kiers et al., 2011). After inoculation, we grew plants for 12 weeks under three atmospheric CO_2_‐levels: low CO_2_, ambient CO_2_, or elevated CO_2_ (see Section 2.2). We used a total of ten replicates per treatment, that is, a total of 120 plants (3 CO_2_‐levels * 4 AMF‐treatments).

### 2.2 Plant growth conditions

First, we scarified and sterilized *M. truncatula* seeds using 95% H_2_SO_4_ for 6.5 min, rinsing them six times in an excess of demineralized water to remove all traces of acid. The scarified seeds were cold‐treated at 4°C for 4 days and then planted in autoclaved peat‐based germination mix. After 10 days, we washed the seedling roots with demineralized water to remove the germination mix. We then transferred the seedlings to sterilized pots (max. volume 662 ml, type MXC12, Pöppelmann, Lohne, Germany) containing autoclaved quartz sand (≥99.5% SiO_2_). Every 2 weeks, we added 25 ml of Hoagland solution per pot (Hoagland & Arnon, 1950) with P content reduced to 50% of the standard solution and N content increased to 150% to favor mycorrhizal colonization (Johnson, 2010). Plants were grown in fully controlled climate chambers at Utrecht University, under a 12‐/12‐hrs day/night regime, 22/17°C day/night temperature and 70% air humidity and were regularly watered. Light intensity during the day was 315 μmol m^−2^ s^−1^ (*SD* 14). Plants were divided into three CO_2_‐controlled climate chambers (Reftech B.V., Sassenheim, the Netherlands) which recorded the following average CO_2_ levels during the 2 months of growth: low (161 ppm, *SD* 7.5), ambient (496 ppm, *SD* 58), or elevated (743 ppm, *SD* 73) CO_2_‐levels. While access to additional CO_2_ chambers would have allowed us to further randomize the plants across chamber, we were limited to a single chamber per CO_2_ treatment, a common limitation in CO_2_ manipulation studies (Field et al., 2012; Kohler et al., 2010; Temme et al., 2015). Within each CO_2_‐chamber, plant locations were fully randomized to account for within‐chamber variation.

### 2.3 Arbuscular mycorrhizal fungal inoculation

We followed the same AM fungal inoculation procedure as previously described (Werner & Kiers, 2015b), suspending root organ cultures of our two AM species in demineralized water to collect spores, and standardizing to densities of 250 spores/ml (Engelmoer, Behm, & Kiers, 2014; Werner & Kiers, 2015b). At planting, we randomly assigned seedlings an AM‐treatment and CO_2_‐level and applied a suspension volume corresponding to 1,000 spores of *R. irregularis*,* G. aggregatum* or a 1:1 mix of both species directly to the roots. For the negative control plants, we applied the same amount of demineralized water (4 ml).

### 2.4 Harvest protocol and intraradical AM fungal abundance

We destructively harvested all plants 12 weeks after planting, following the same harvest protocol as described previously and determined plant aboveground dry weight (Werner & Kiers, 2015b). We cut each individual root system in small fragments (~1 cm) and divided it in three randomized root fragment subsets: One was frozen at −20°C and used for later molecular analyses, one subset was stored in individual plastic bags at 4°C and used to inoculate a next generation of plants in the mixed AM treatments, and the third subset was used to determine belowground dry weight. In order to obtain belowground dry weight, we immediately weighed the full belowground fresh weight and the third root subset fresh weight. We then determined the subset's dry weight and used the ratio of dry to fresh weight to calculate full belowground dry weight for each plant. Two plants per each of the three mycorrhizal treatments died during the experiment under low CO_2_‐conditions. We removed these from our analyses; consequentially, there are only eight replicates in all low CO_2_‐conditions inoculated with AM fungi.

To determine intraradical AM fungal abundance, we used quantitative PCR following the same protocol as previously described for these AM species and host plants (Engelmoer et al., 2014; Werner & Kiers, 2015b). Briefly, we used primers specific to *G. aggregatum* and *R. irregularis*, allowing us to discriminate and quantify intraradical abundance of both species even when present in a mixed inoculum (Engelmoer et al., 2014; Kiers et al., 2011). AM fungal abundances as measured with this exact same protocol have a strong positive correlation (Pearson's *r* = .58) with microscopic AM colonization scoring (Werner & Kiers, 2015b) as well as with extraradical fungal biomass (Pearson's *r* = .81) (Engelmoer et al., 2014), but visual identification cannot discriminate these species when colonizing the same root systems as in this study. We therefore analyzed AM fungal abundance as expressed in copy numbers per mg freeze‐dried roots, as previously correcting for DNA extraction efficiency of each sample (Engelmoer et al., 2014; Werner & Kiers, 2015b).

### 2.5 Multigenerational transfer of AM fungi

Using the same inoculation and plant growth conditions as for our first generation of plants, we inoculated a new generation of *M. truncatula* seedlings using an average of 1.35 g (*SD* 0.23) of mycorrhizal root fragments and 61 g (*SD* 14.2 g) of soil from our mixed AM fungal treatments. This allowed us to transfer spores in the soil, and on the mycorrhizal root fragments, thus colonizing the new generation of plants. Following previous work (Verbruggen et al., 2012), this transfer protocol simulates the process occurring in the field when a new generation of annual plants is recolonized by AM fungi from infected roots and soil spores, allowing us to study potential long‐term shifts in AM species composition in a greenhouse setting. Using this technique, we grew plants for 12 weeks in the same controlled CO_2_ climate chambers before destructively harvesting them, and analyzing them as previously. AM fungi were not pooled between generations but were propagated independently for each replicate plant. We studied a total of three plant generations. Plants at each generation were grown from the same batch of seeds, so we only observe shifts in the mycorrhizal community, not evolutionary responses of the host.

To determine if this transfer protocol of fungi to subsequent generations of host plants was equally efficient for both species, we performed an additional study of AM transfer across two generations for both species inoculated in monoculture. Our aim was to ensure that changes in relative abundances over generations were not caused by differences in transfer efficiency, for instance due to a lower disturbance resistance of one AM species compared to another. This pilot experiment revealed that for both *G. aggregatum* and for *R. irregularis*, AM fungal abundance actually increased between two test generations (Figure S1, *F*_1,36_ = 41.97, *p* < .01). We also found that *R. irregularis* had a significantly higher overall abundance than *G. aggregatum* (*F*_1,36_ = 0.05, *p* = .01), but we found no significant interaction term between generation and AM‐treatment (*F*_1,36_ = .05; *p* = .63), statistically confirming that there were no differences in transfer efficiency between the two AM species, and that AM fungi can be maintained and even increase in abundance between plant generations using this protocol (Figure S1).

### 2.6 Statistical analysis

We performed all our statistical analyses in R 3.4.1. All data have been archived, and we provide an R‐script to replicate our analyses and figures on the Dryad repository (https://doi.org/10.5061/dryad.2kj8p). We first analyzed full plant dry weight in the first generation to determine how CO_2_‐level and AM‐inoculation affect plant growth. We generated a linear model of the effects on full plant dry weight of CO_2_‐level, *G. aggregatum* presence and *R. irregularis* presence, and their two‐way interactions. This enabled us to test for the effect of presence of either AM fungus on plant growth, allowing us to estimate if, as in previous research (Kiers et al., 2011), *R. irregularis* was a higher quality partner then *G. aggregatum*. To evaluate if plant growth was affected by abundance of either AM fungus, we additionally analyzed a linear model of the effects of CO_2_, *G. aggregatum* abundance, *R. irregularis* abundance and their interactions on full weight of the plants inoculated with both fungi. Second, we studied the effect of CO_2_ on the intraradical AM fungal abundance when plants were inoculated with monocultures of each AM species, using ANOVA‐models for both AM species and the three CO_2_‐levels as explanatory factors. This allowed us to determine if these fungal species could successfully colonize plants under the CO_2_‐conditions used. Third, to address our main hypothesis, we analyzed the relative performance over three generations of both AM fungi when grown from mixed inocula on the same root system. We aimed to determine if over time, and across CO_2_‐levels we would observe a relative increase of *R. irregularis* in the mixed AMF communities. To test this, we calculated the log response ratio of the abundances of both fungi log (*R. irr*/*G. agg*) This is a metric of the relative success of both fungi, with higher positive values indicating a relatively higher abundance of *R. irregularis* and negative values indicating *G. aggregatum* being more successful in colonizing plant roots (Hedges, Gurevitch, & Curtis, 1999; Hoeksema et al., 2010; Konvalinková & Jansa, 2016). We generated a linear model of this metric as response variable, with CO_2_‐level and generation as explanatory variables, allowing us to test the relative performance of both fungi across generations and CO_2_‐levels, including the potential for shifts over time in the relative success of the higher quality AM fungi. We used R‐package *phia* to perform post hoc analyses of CO_2_‐level effects within generations (De Rosario‐Martinez, 2015).

**Figure 1:**
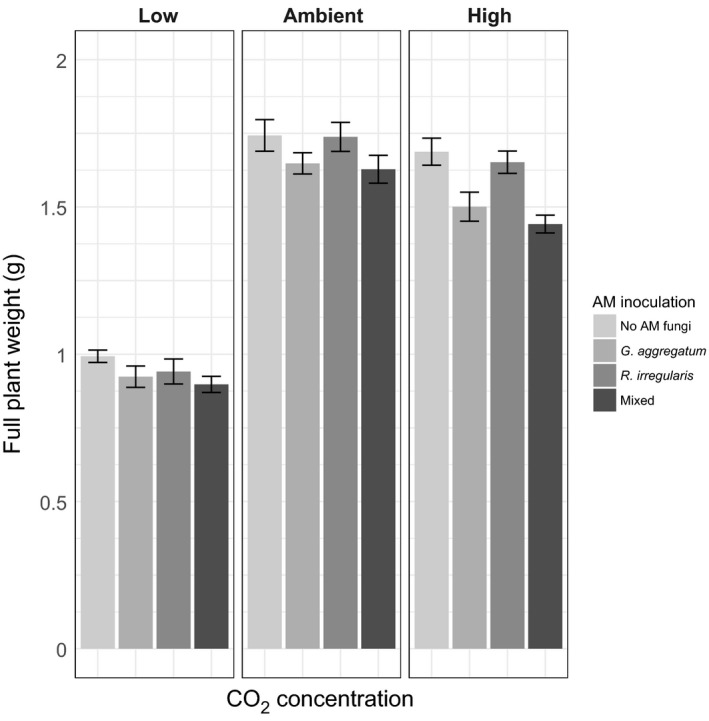
Full plant weight (g) for each arbuscular mycorrhizal (AM) fungi treatment at each CO_2_‐level (±*SE*). Panels indicate CO_2_‐level plants were grown in (low, ambient, and high), colored bars indicate AM fungal inoculation (No inoculation, monoculture of *Glomus aggregatum* or *Rhizophagus irregularis*, or mixed inoculation). Total *N* = 113

In all our analyses, we set AM fungal copy numbers that were below the limit for reliable detection to equal the detection limit (Engelmoer et al., 2014; Werner & Kiers, 2015b). This means that samples where AM fungal abundance was extremely low were analyzed as if the abundance was at the lower limit for reliable quantification of AM fungal abundance. In our analyses of multigenerational AMF abundances, we observed samples below the detection limit in 41 cases for *G. aggregatum*, and in zero cases for *R. irregularis*. This procedure makes our analyses more conservative, because it makes it impossible to observe complete exclusion of *G. aggregatum* (which turned out to be the lower quality AM species, Figure 1) from roots, and effectively overestimates its abundance and relative success.

## 3 RESULTS

### 3.1 CO_2_‐level and AM fungi inoculation influence plant growth

We generated a linear model of plant growth in the first generation, with *G. aggregatum* and *R. irregularis* inoculation as separate binary factors, and CO_2_‐level a three‐level factor. This enabled us to determine differences in partner quality by testing if and how inoculation with either fungus affects plant growth across treatments. If *G. aggregatum* is a low‐quality partner and *R. irregularis* is a higher‐quality partner, we expect significantly negative or neutral growth effects of the former, and positive effects of the latter. In agreement with the first expectation, we found that inoculation with *G. aggregatum* significantly reduced plant growth (Mean reduction in plant biomass when inoculated with *G. aggregatum* 0.10 g; *F*_1,103_ = 18.70, *p* < .01; Figure 1). However, inoculation with *R. irregularis* did not significantly increase or decrease plant growth (Mean reduction when inoculated with *R. irregularis*: 0.01 g; *F*_1,103_ = 0.23, *p* = .63). Lastly, we found no interaction among *G. aggregatum* and *R. irregularis* inoculation (*F*_1,103_ = 0.04, *p* = .85), meaning that the effect of *G. aggregatum* on plant growth is independent of the effect of *R. irregularis* on plant growth, and vice versa. These results confirm that *R. irregularis* is a higher‐quality partner than *G. aggregatum,* but reveal that neither fungal partner was beneficial to their host plants under these growth conditions.

We also found a significant overall effect of CO_2_‐level (*F*_2,103_ = 366.59, p < .01) on plant biomass, but no interaction of CO_2_‐level with either *G. aggregatum* (*F*_2,103_ = 2.99, *p* = .05) or with *R. irregularis* inoculation (*F*_2,103_ = 0.21, *p* = .81). This indicates that AM‐effects on plant growth were not mediated by CO_2_‐levels. As predicted, we found that plants grown under low CO_2_ were smaller than under ambient CO_2_ (Figure 1; ambient CO_2_ mean weight 1.69 g ± 0.02 *SE*; low CO_2_ mean weight 0.94 g ± 0.02 *SE*;* p* < .01). However, in contrast to our expectations, we found that plants grown under elevated CO_2_‐levels were slightly smaller on average than under ambient (elevated CO_2_ mean weight 1.57 g ± 0.03 g; *p* < .01).

We also set out to establish if, in inoculated plants, there is a correlation among fungal abundance for either AM fungus (copy number per mg root) or plant biomass. To evaluate the effects of both fungi, we analyzed the plants inoculated with a mix of both AM fungi, but we found that only CO_2_‐level (*F*_2,16_ = 77.38, *p* < .01) and not abundance of *R. irregularis* (*F*_1,16_ = 0.72, *p* = .41; Figure S2) or *G. aggregatum* (*F*_1,16_ = 0.44, *p* = .52; Figure S3) drove full plant dry weight of colonized plants (Table S1). These results suggest that while there is an overall effect of inoculation with AM fungi on plant growth (Figure 1), the effect does not depend on the colonization level established by the AM fungi.

### 3.2 Both fungi benefit from increasing CO_2_‐levels

**Figure 2:**
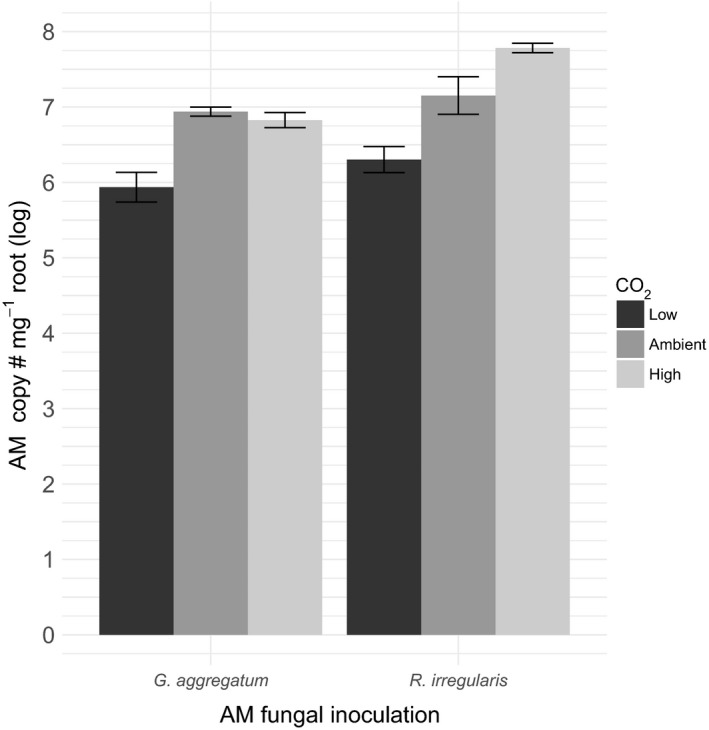
Mean intraradical fungal root abundance (logarithms of copy number per mg dry root mass, ±*SE*) for both *Glomus aggregatum* and *Rhizophagus irregularis*. Plants were inoculated with a monoculture of either arbuscular mycorrhizal fungus (indicated on the *x*‐axis) at three CO_2_‐levels. Total *N* = 56

We then tested if root colonization by each fungal species was affected by CO_2_‐levels when grown in monoculture. We found that fungal abundance increased with increasing CO_2_‐levels (Figure 2), both for *R. irregularis* (*F*_2,25_ = 15.98, *p* < .01) and for *G. aggregatum* CO_2_ (*F*_2,25_ = 18.93, *p* < .01). These results confirm that both AM fungi can establish themselves in the roots at all CO_2_‐levels tested, and that generally fungi benefit from increasing CO_2_‐levels, potentially due to the increased availability of carbon.

To further analyze the potential role of plant preferential rewarding under different CO_2_‐levels, we determined the relative success (log response ratio of fungal abundances) for both the single treatments (no potential for rewarding) and the mixed treatment (potential rewarding). As plants were not paired in the single treatments, we cannot calculate this at the level of the individual plant but only averaged across CO_2_‐treatments. Yet, we find that the direction of the effect is as expected with *R. irregularis* doing substantially and increasingly better with increasing CO_2_‐level when there are potential preferential allocations (Table 1).

**Table 1:** Mean relative AM fungal success and potential for preferential allocations

CO_2_	Mixed (with potential host preference)	Single (no host preference possible)
Low	0.58	0.37
Ambient	1.32	0.21
High	2.08	0.96

Relative success of the higher‐quality arbuscular mycorrhizal (AM) fungus *Rhizophagus irregularis* is defined as log (*R. irr*/*G. agg*), thus higher values indicate relatively more successful *R. irregularis*. Mixed treatment relative success is based on data for the first generation, to maximize comparability with the single treatments.

### 3.3 Plants reduce the colonization of low‐quality fungi over multiple generations and with increasing CO_2_‐levels

**Figure 3:**
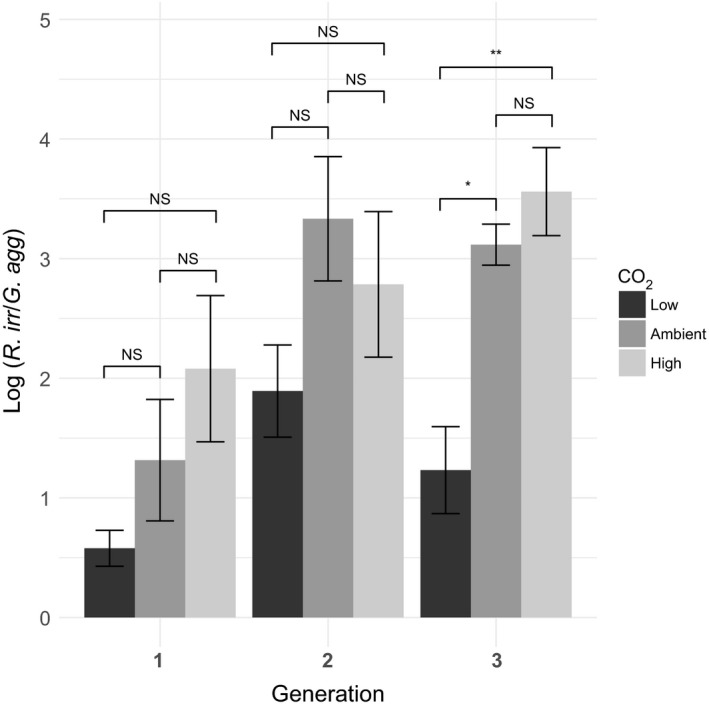
Mean relative success (log(*R. irr*/*G. agg*) ± *SE*) of the two AM species when grown in a mix on the same root system over three generations. The *x*‐axis indicates the three successive plant generations, the colored bars indicate CO_2_‐levels plants were grown under. Positive values indicate that *Rhizophagus irregularis* has a higher root abundance than *Glomus aggregatum*, negative values would indicate the reverse. The more positive, the more successful *R. irregularis* is relative to *G. aggregatum*. Total *N* = 84

We then analyzed the relative abundance of each fungus when grown in the other's presence on a single root system (log *R. irregularis*/*G. aggregatum*). We find that in all cases, its mean value is in the positive domain, indicating a higher relative abundance of *R. irregularis* than of *G. aggregatum*, and that this increases over the generations, and with CO_2_ level (Figure 3). Testing for the effect of atmospheric carbon and generation on relative AM fungal abundance, we found a significant effect of both CO_2_‐level (*F*_2,75_ = 8.77, *p* < .01) and plant generation (*F*_2,75_ = 9.61, *p* < .01), but not of its interaction (*F*_4,75_ = 1.06, *p* = .38). When we tested for CO_2_‐effects within generations, we found that after three generations, *R. irregularis* relative abundance was higher in ambient CO_2_ and in elevated CO_2_‐environments compared to in low CO_2_ (respectively, *p* = .045 and *p* = .01), while prior to that there was no significant effect of CO_2_ within generations. This suggests it takes at least three generations for the higher‐quality AM fungus to do significantly better in the two higher CO_2_‐levels compared to in low‐CO_2_ conditions.

## 4 DISCUSSION

We aimed to test if external resource availability affects how plant hosts mediate carbon allocations to fungal partners that vary in the benefit they provide to their hosts. We found that over three plant generations, AM fungal communities of host roots became more dominated by the higher‐quality fungus, *R. irregularis* (Figure 3). This supports our expectation that as CO_2_ increases, plant hosts more efficiently allocate resources to higher quality AM fungi, resulting in an increasingly less harmful fungal community over generations [hypothesis (i)]. In contrast, our results do not support a scenario where plant become less selective in their partner choice with increased carbon (Kiers & van der Heijden, 2006), or where plant allocation to AM fungi is a fixed response independent of context. Specifically, we found that after three generations, *R. irregularis* was more successful in plant hosts grown in elevated and in ambient CO_2_‐conditions compared to in plants grown in depressed CO_2_. This reveals that loss of lower‐quality AM fungi from the population requires time and is affected by CO_2_‐level. Our results suggest that (1) atmospheric carbon levels can influence plants’ ability to favor higher‐quality AM species, (2) that increasing CO_2_‐levels drive more stringent partner choice, (3) that in the long run, low CO_2_‐levels may help less cooperative AM fungi spread in the population.

Our findings are consistent with predictions that environmental conditions, and specifically the relative availability of exchanged resources, affect plant host capacity to structure its symbiotic community (Bever, 2015; Wyatt et al., 2014). Previous experiments have shown that reducing plant carbon budgets by shading, diminishes plant preferential allocations to more beneficial AM partners (Zheng et al., 2015). This is similar to our finding that the relative success of a higher‐quality AM fungus falls under low‐CO_2_ conditions (Figure 3). The likely reason for this effect is that as carbon becomes more restricted, the relative value to the plant of AM‐provided soil nutrients falls, reducing the incentive for stringent selection of higher‐quality AM partners (Bever, 2015; Wyatt et al., 2014). Our results now reveal that successful reduction of the abundance of the low‐quality AM partner in depressed CO_2_ conditions compared to higher CO_2_ levels can take time, in our case three *M. truncatula* generations (36 weeks). This highlights another theoretical prediction: That CO_2_‐effect on plant preferential reward mechanisms is relatively weak and may only appear when measured over considerable time (Wyatt et al., 2014). An open question now remains how plant choice operates over different time scales when mycorrhizal networks connect multiple plant hosts that differ in relative carbon availability. A study where a mycorrhizal network was simultaneously connected to a shaded and an unshaded plant, found that in a single plant generation (8 weeks) the higher‐quality AM fungus performed relatively better in shaded than in unshaded plants (Knegt et al., 2016), in contrast to our results here. Potentially, carbon acquired from the unshaded plant allowed the higher‐quality AM fungus to outcompete its competitor in the shaded plants (Knegt et al., 2016). While in the current study, we focus on the potential for partner choice by a single plant, this highlights that fungal colonization dynamics may be affected by the wider mycorrhizal network in which it is embedded.

We emphasize that we did not directly measure nutrients flows, but only fungal abundance patterns. While our results are consistent with dynamic changes in host‐directed benefits to mycorrhizal symbionts, we cannot exclude a role for direct competition between AM fungi, either within roots or within the soil (Engelmoer et al., 2014; Hepper et al., 1988; Kennedy, 2010). However, this would assume that CO_2_‐level directly impacts competition among the two AM species through other means than via plant allocations. This is unlikely because AM fungi do not have direct access to environmental carbon, but only via plant mediation (Parniske, 2008). Additionally, our results show that in monocultures, both species show similar responses to varying CO_2_‐levels (Figure 2), suggesting an absence of different direct effects of CO_2_ on AM colonization dynamics. The importance of preferential choice mechanisms relative to other potential (ecological) drivers of CO_2_‐effects now remains an open question.

Preferential allocation strategies are thought to stabilize mutualisms, and limit the spread of low‐quality partners throughout populations and over time (Bever, 2015; Ghoul et al., 2014; Oono, Anderson, & Denison, 2011; Steidinger & Bever, 2014). A major open question is therefore if variation in allocation strategies will ultimately affect long‐term success of competing symbionts over multiple generations. In line with the idea that increasing plant carbon budgets favor the long‐term spread of higher quality fungal partners providing more host benefits, AM inocula from long‐term FACE (Free‐Air Concentration Enrichment) CO_2_‐enriched plots provide more nitrogen to hosts plants (Gamper, Hartwig, & Leuchtmann, 2005). Furthermore, recent work showed that elevated CO_2_ resulted in phylogenetic clustering of AM fungal communities, argued to be consistent with altered host selection for more beneficial fungal partners under elevated CO_2_ (Mueller & Bohannan, 2015). Our results now show that depressed atmospheric carbon reduces the relative success of a higher‐quality AM fungus (Figure 3), suggesting that in the long run, lower‐quality fungi could more effectively spread in these conditions. A potential implication of these various results is that global change, via increased CO_2_ and reduced plant carbon limitations allowing more stringent preferential allocations, may have positive effect on arbuscular mycorrhizal cooperation with plants. However, a multigenerational study of AM communities found that less beneficial AM taxa (such as *Gigaspora* and *Scutellospora* species) were lost under increased CO_2_, but only when the CO_2_‐increase was abrupt (Klironomos et al., 2005). In contrast, under a gradual increase in CO_2_‐level over 6 years, less beneficial AM fungi were retained (Klironomos et al., 2005). This suggests that our experiments may be less suitable as a general model for shifts in AM community composition under more gradual changes in CO_2_‐levels.

For all our analyses, the CO_2_‐effects we observed were strongest when comparing the low CO_2_‐treatment with the other two levels, while ambient and high CO_2_ showed very similar plant growth (Figure 1) and AM colonization patterns (Figures 2, 3). This is likely driven by the fact that while low CO_2_ resulted in substantial plant growth reduction, our elevated CO_2_‐treatment did not increase plant growth compared to ambient CO_2_ (Figure 1). This suggests that in the growth conditions we used, when increasing CO_2_ from ambient to elevated CO_2_, *M. truncatula* was limited by another factor than CO_2_, while over the depressed to ambient domain, CO_2_ was actually a limiting factor. One idea is that the effects of CO_2_ on *Medicago* are temperature‐sensitive. The closely related host plant *Medicago sativa* was found to only benefited from elevated CO_2_ when temperature was also elevated (4°C increase from standard 19°C) (Aranjuelo et al., 2008). Future studies can now further test if our conclusions also hold over ambient to elevated CO_2_‐increases by analyzing environmental conditions (such as higher temperatures or higher light intensity) where higher atmospheric carbon actually increases plants’ carbon budgets. A second limitation is the low to negative effects of AM fungi on plant growth found under our laboratory conditions (Figure 1). Potentially, plants did not experience general positive fitness benefits from fungal inoculation due to relatively short day lengths and light intensities of our growth chambers. While the growing conditions still allowed us to test the relative difference between symbionts (Figure 1), extending our studies to include multiple AM species and environmental conditions, including conditions that induce positive growth benefits would allow us to better study the long‐term effects of CO_2_‐level on plant‐mycorrhizal cooperation.

Our work illustrates how environmental context can affect the extent to which organisms structure interactions with their mutualistic partners. We suggest that mechanisms evolved to limit the spread of low‐quality partners are sensitive to changing different environmental conditions. An open question is to what extent variation in the strength and precision of partner choice mechanisms across different species and across different mutualisms are driven by contemporary or historical environmental variation (Grman, 2012; Jandér & Herre, 2010; Jandér et al., 2012; Oono, Denison, & Kiers, 2009; Werner & Kiers, 2015a). For instance, in the plant‐rhizobial mutualism, sanction strength for less cooperative rhizobia was found to both be affected (Kiers, Rousseau, & Denison, 2006) and not directly affected by fertilization (Regus et al., 2014), showing that environmental effects on strength of partner choice may not be uniform. One idea is that over evolutionary time, ecological conditions where preferential allocations are less effective, could select for the loss of such mechanisms, potentially in turn decreasing the level of symbiont cooperation (Simonsen & Stinchcombe, 2014; Steidinger & Bever, 2014). A particularly promising model of this dynamic may be the potential loss of partner choice and mutualism in response to host plant domestication (Kiers, Hutton, & Denison, 2007; Xing et al., 2012). More generally, we predict that environmental conditions that reduce the relative value to an organism of mutualistically provided services or resources cause reduced selection for stringent partner choice mechanisms, resulting in a potential degradation of these mechanisms and in relative increase in lower‐quality partners in those habitats.

## CONFLICT OF INTEREST

None declared.

## AUTHOR CONTRIBUTIONS

GDAW, CMJP, and ETK designed research. GDAW and YZ performed the experiments. GDAW performed the analyses. GDAW and ETK wrote the manuscript. All authors contributed to revised versions of the manuscript and approved its submission.
