# Supplementary material for: Tracking plant preference for higher‐quality mycorrhizal symbionts under varying CO 2 conditions over multiple generations
Source: Ecol Evol. 2017 Nov 23;8(1):78–87. doi: 10.1002/ece3.3635 (PMC5756855; doi:10.1002/ece3.3635)
Supplement: Supplementary file 1 [file ECE3-8-78-s001.docx]

*
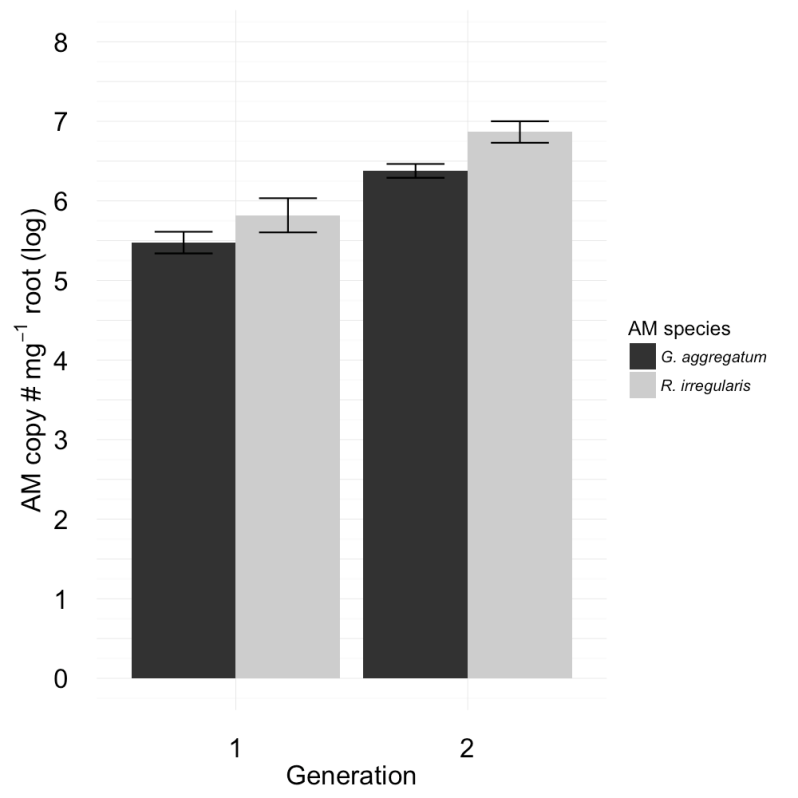
*

*Figure S1: Mean intraradical AMF root abundance (copy number mg^-1^ dry root mass, ± S.E.) for both AM species across two generations (total N=40).*

*
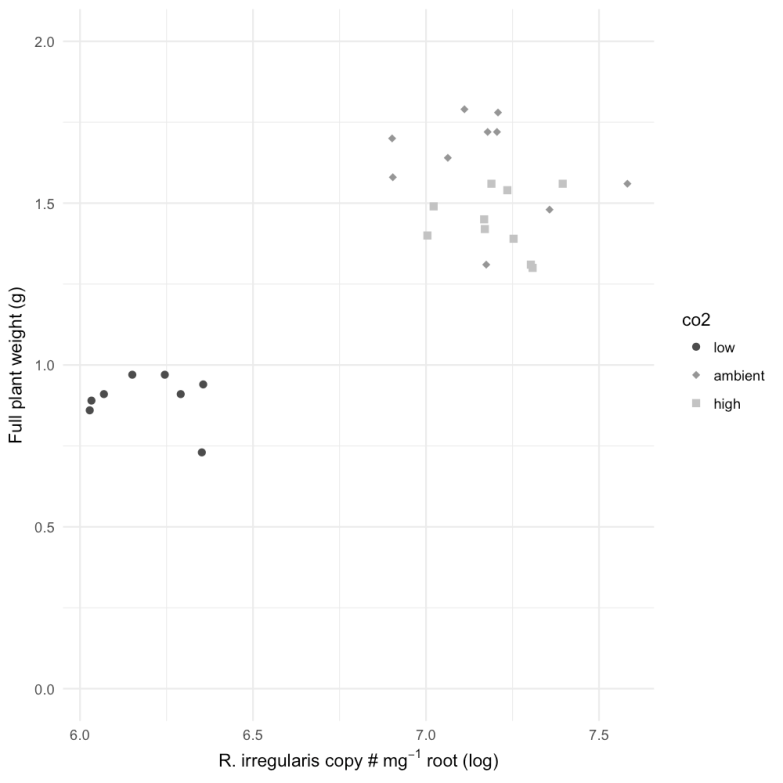
*

*Figure S2: Full weight of plants inoculated with both fungi and R. irregularis copy number (Total N = 28).*

*
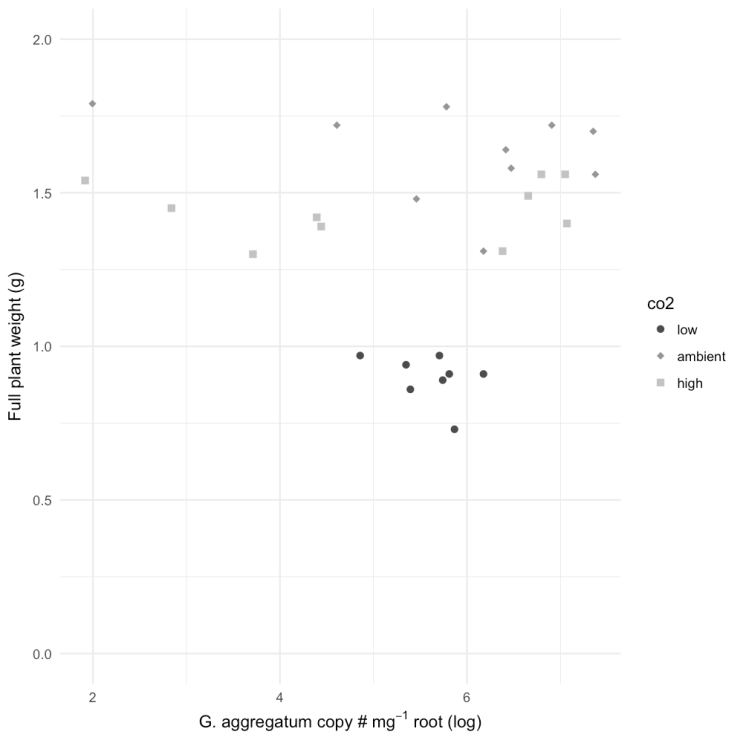
*

*Figure S3: Full weight of plants inoculated with both fungi and G. aggregatum copy number (Total N = 28).*

| **Table S1: ANOVA-table full plant weight** | | | | | |
| --- | --- | --- | --- | --- | --- |
| Factor | Df | SS | MS | F-value | P |
| CO_2_ | 2 | 2.50 | 1.25 | 77.38 | **< 0.01 ***** |
| *G. aggregatum* abundance (copy nr) | 1 | 0.01 | 0.01 | 0.44 | 0.52 |
| *R. irregularis* abundance (copy nr) | 1 | 0.01 | 0.01 | 0.72 | 0.41 |
| *G. agg* : CO_2_ | 2 | 0.02 | 0.01 | 0.63 | 0.54 |
| *R. irr* : CO_2_ | 2 | 0.00 | 0.00 | 0.06 | 0.94 |
| *G. agg* : *R. irr* | 1 | 0.01 | 0.01 | 0.76 | 0.40 |
| *G. agg* : *R. irr* : CO_2_ | 2 | 0.01 | 0.01 | 0.46 | 0.64 |
| Residuals | 16 | 0.26 | 0.02 |  |  |
| *The above give the ANOVA-table for a linear model analysing the effect on full plant dry weight of CO_2_-condition, R. irregularis abundance and G. aggregatum abundance, as well as all potential interactions. The associated data are represented in Figures S2 and S3.* | | | | | |
